# Advanced image preprocessing and context-aware spatial decomposition for enhanced breast cancer segmentation

**DOI:** 10.1016/j.mex.2025.103224

**Published:** 2025-02-15

**Authors:** G. Kalpana, N. Deepa, D. Dhinakaran

**Affiliations:** a Department of Computer Science and Engineering, Saveetha School of Engineering, Saveetha Institute of Medical and Technical Sciences, Chennai, India; b Department of Computer Science and Engineering, Vel Tech Rangarajan Dr. Sagunthala R&D Institute of Science and Technology, Chennai, India

**Keywords:** Breast cancer, Segmentation, Normalization, Augmentation, Equalization, Multi-scale region enhancement, AIPT (Advanced Image Preprocessing Techniques) and CASDN (Context-Aware Spatial Decomposition Network)

## Abstract

The segmentation of breast cancer diagnosis and medical imaging contains issues such as noise, variation in contrast, and low resolutions which make it challenging to distinguish malignant sites. In this paper, we propose a new solution that integrates with AIPT (Advanced Image Preprocessing Techniques) and CASDN (Context-Aware Spatial Decomposition Network) to overcome these problems. The preprocessing pipeline apply bunch of methods including Adaptive Thresholding, Hierarchical Contrast Normalization, Contextual Feature Augmentation, Multi-Scale Region Enhancement, and Dynamic Histogram Equalization for image quality. These methods smooth edges, equalize the contrasting picture and inlay contextual details in a way which effectively eliminate the noise and make the images clearer and with fewer distortions. Experimental outcomes demonstrate its effectiveness by delivering a Dice Coefficient of 0.89, IoU of 0.85, and a Hausdorff Distance of 5.2 demonstrating its enhanced capability in segmenting significant tumor margins over other techniques. Furthermore, the use of the improved preprocessing pipeline benefits classification models with improved Convolutional Neural Networks having a classification accuracy of 85.3 % coupled with AUC-ROC of 0.90 which shows a significant enhancement from conventional techniques.

• Enhanced segmentation accuracy with advanced preprocessing and CASDN, achieving superior performance metrics.
• Robust multi-modality compatibility, ensuring effectiveness across mammograms, ultrasounds, and MRI scans.

## Graphical abstract

**Figure:**
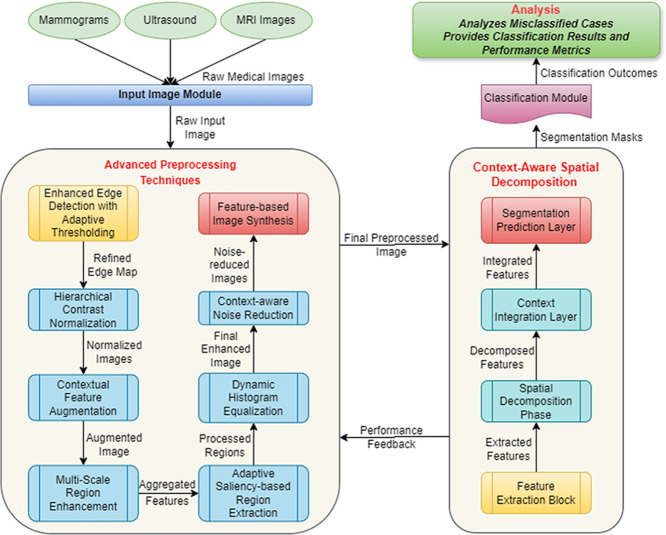

Specifications table

**Table:** 

Subject area:	Engineering
More specific subject area:	Breast Cancer Segmentation
Name of your method:	AIPT (Advanced Image Preprocessing Techniques) and CASDN (Context-Aware Spatial Decomposition Network)
Name and reference of original method:	Dynamic Machine Learning Approach, Enhanced Image Contrast and Feature Extraction
Resource availability:	N*.A.*

## Background

### Introduction

Breast cancer is a common type of cancer among women globally and stands as a major killer through cancer related diseases. This is because early discovery of the disease and correct diagnosis is key determiner to better prognosis as it is well known that chances of this disease advancing increase with time. Mammography, ultrasound and MRI are the examples of imaging diagnostics that can help to pinpoint the location of cancerous tissues [1]. The image segmentation is crucial in identifying regions of interests, such as tumor margin, morphology and malignant/normal regions. However, breast cancer image segmentation still presents a challenging problem because of the inherent characteristics and complexity of medical images. Breast imaging is one of the major challenges when it comes to diagnosing breast cancer since the image quality is fairly variable [2]. Aspect like contrast variations, different levels of brightness and the presence of noise will all reduce the quality of images needed in the diagnosis of certain conditions. These problems often contribute to the inform precision in delineation of tumor margins and make escalation of segmentation impractical. Not only that, images of breast cancer often have a low resolution and low contrast or it is difficult to distinguish micro textures between cancerous and non-cancerous tissues [[3], [4], [5]]. These challenges stem from differences in the types of imaging equipment used, the patients being studied, and the conditions under which scans are performed making it essential that optimal and portable techniques are employed.

These challenges have, for a long time, made use of traditional image segmentation methods such as Otsu's thresholding, region-growing techniques, and watershed algorithms. These methods are based on certain pre-determined value, or pixel intensity value to segment the images [6]. Although these techniques are accurate and perform satisfactorily in simplified test environments where image characteristics are known in advance, they do not always transfer well from one data set to another because they are unable to adapt to changes in circulating image attributes on the fly. Support Vector Machines (SVMs) and random forests are few machine learning solutions that have at least offered an automated and dynamic mode of operation. But they are dependent on the quality of input variable and if necessary extensive variable construction process has to be performed to get feasible outcomes of the modes [[7], [8], [9]]. In recent years, deep learning techniques especially CNN and encoder-decoder networks including U-net and Deep Lab, have shown considerable potential for breast cancer image analysis. A great feature of these models is that they are able to work well with more complicated spatial characteristics, and in the case of this research, segmentation precision has been proved to have been enhanced greatly. However, they rely on the quality of input images; large annotated datasets for training are also required. Poor preprocessing often causes deep learning models to fail to segment small details, which are critical for proper segmentation. Moreover, existing methods do not possess features allowing the reconstruction of substantially important contextual information that is valuable to precisely outline a tumor shape and locate the regions of interest [10].

To address these issues, we introduce an integrated system that includes Advanced Image Preprocessing Techniques and the innovative Context-Aware Spatial Decomposition Network (CASDN). The preprocessing system we propose is an effective solution for the main issues of breast cancer imaging by improving image quality, edge detection, contrast standardization, and feature expansion. Special emphasis of the preprocessing techniques is given to adaptive edge detectors, hierarchal contrast normalization, contextual features expansion, multi-scale region enhancement, saliency-based region extraction, dynamic histogram equalization, context-guided noise removal, and synthesizing features based on images. These steps can be seen as preparing input images to be fed to the CASDN framework – the corresponding changes aim at enhancing the clarity, or reducing variability of input images which enhances their CASDN framework performance. This advantage progresses further in the CASDN framework through refined spatial feature decomposition and contextual information fusion. Unlike traditional segmentation models, CASDN incorporates a dual-stage approach: In order to perform the segmentation, it effectively extracts and analyses local pixel level features and structural features along with a systematic understanding of the image context. This makes the network capable of flexibly maintaining high performance on any given set of images while its size adapts to differences in the image content and size. The synergy accorded by preprocessing and segmentation makes it easy for the framework to achieve optimal boundary detection, feature extraction as well as segmentation.

The proposed framework overcomes the existing approach limitations by proposing the new technique to enhance the breast cancer prediction accuracy. To show the superiority of our framework, we compare it with multiple baseline models and traditional algorithms which demonstrate its improved performance based on accuracy, precision, recall and F1 score. At each stage, we monitor the segmentation performance by reporting the Dice coefficient, Intersection over Union (IoU), confidence, accuracy and the Hausdorff distance, proving that our method can accurately extract the detailed part for the tumor region.

### Related work

The field of breast cancer detection has evolved significantly over the last two decades due to increasing concern with developing improved technologies to provide accurate diagnoses and thereby help to cut breast cancer deaths. Breast cancer is still ranked high among the prevalent cancer diseases and is among the leading causes of cancer related fatalities in women. However, there are still barriers to early and precise diagnosis even in the present era of advanced health-care systems and diagnostics procedures. This section presents a critical discussion of current research on breast cancer detection methods with special emphasis on the traditional methods and their restrictions, as well as new developments in the field. Eltalhi et al. [11] proposed to enhance the quality of the ultrasound data of breast cancer through the preprocessing method on the home medical dataset. All of them used regression imputation and Synthetic Minority Over-sampling Technique (SMOTE) to solve the missing values and data imbalance problems and obtained three different datasets besides the original one. In the process of the feature selection, CFS and ReliefF methods were used. Although their method was useful and accurately dealt with data quality and imbalance problems, they focused only on one database and particular preprocessing techniques, which may reduce the transferability of their conclusions to various imaging modalities.

Fadida-Specktor et al. [12] put forward a dynamic machine learning approach for predicting the right advanced visualization algorithm from the free text DICOM tags. Their system was comprised of a Bag of Words (BOW) feature extractor and a Random Forest classifier. While this approach benefitted from sophisticated feature extraction and classification schemes, this work's focus on textual metadata means that important aspects of imaging data might be missed. In this research, Rani et al. [13] examined how various preprocessing filters such as gaussian, median, and bilateral preprocessing filters affects image fusion with the CNNs. According to findings that were presented by Arakelian et al., the median filter was the most effective all through the groups of images. However, their study was restricted to examine only the effects of filter methods and did not compare other methods of preprocessing along with aggregate methods, nor investigate the fusion of preprocessing, feature selection and classification procedures. Raad et al. [14] provided a systematic evaluation of 24 preprocessing configurations on three clinical datasets. They evaluated normalization, the method of region of interest selection, bias field correction and resampling techniques. While their integration allowed to consider a large number of successful variants of configurations and reveal the effectiveness of different types of preprocessing techniques, the certain selection of cases may weaken the effect of each method on particular types of imaging data. Bozhenko et al. [15] addressed about issues with data preprocessing in medical systems and more specifically no or null values, and anomalous and outlier values. They gave a brief introduction about preprocessing stages and some preprocessing techniques like histograms and box plots for outlier treatment. Although their approach provided an extensive review on the preprocessing techniques, their study was more general in analyzing data while specific on breast cancer was not well seized. The effects of most frequently applied image preprocessing techniques on MRI radiomic features in glioblastoma collections of data. They found out the impact of preprocessing on feature robustness, though their research did not focus on breast cancer strain and was conducted only for MRI modality. There are authors that pointed out that it is crucial to choose the right preprocessing techniques that may contribute to the increase of classification level.

Almuhaideb et al. [16] focused on data pre-processing methods which they explored include discretization, attribute selection and missing values. Their findings showed how preprocessing affected the classification capability of datasets, though without concentrating on certain forms of imaging. Chalo et al. [17] proposed a method of detection of diabetes using a deep neural network, which employed cross-validation training. While their method was useful in uncovering potential uses of deep learning, it was not applicable to distinguishing between malignant and benign tumors or to preprocessing techniques. Hu et al. [18] put forward an efficient data preprocessing model containing instance selection and model training. Their method is helpful, it does not contain particular methods employed for medical imaging data preprocessing. They have made an attempt to develop a three-phase method that consisted of data preprocessing, feature outlier detection, and feature selection. As for the outlier detection and the feature selection Whale Optimization Algorithm (WOA) they showed that they had a systematic approach and did not focus on breast cancer imaging. Ha Anh et al. [19] have focused on preprocessing to improve those characteristics of images that are material for tumor detection. Gaussian smoothing, bilateral filtering and K-means clustering were also applied to show how preprocessing can enhance the classification outcomes significantly. However, their study failed to incorporate the preprocessing work with other training, feature extraction and classification techniques.

A comparative summary of the existing methodologies in the context of breast cancer segmentation is availed in the above Table 1. A review of existing work in the area of breast cancer detection and segmentation demonstrates important progress made but also important drawbacks that reduce the reliability and the scope of the proposed solutions. Most approaches used today are associated with some imaging types like, ultrasound or mammograms and utilize preprocessing techniques ideal for specific databases limiting the capacity to handle other imaging sources. Furthermore, new approaches, for example, based on Gradient Vector Flow (GVF) and attention-based networks, are introduced but they require a vast number of computations, are sensitive to parameter fine-tuning, and may have dataset-specific limitations. A majority of them focus on individual preprocessing strategies that include filtration, enhancements or some texture analysis techniques; however, do not pay much attention on how these methods can be harvested with reasonable feature extraction/selection and classification models. In addition, there is also a shortage of multi-scale and context aware preprocessing solutions that reduces the opportunity to identify the global structures and detailed features necessary for segmentation. To fill these gaps, the proposed work introduces a novel framework by incorporating an elaborate version of the advanced preprocessing module with the CASDN. Thus, we employ adaptive edge detection using Sobel and Canny edge detector, hierarchical contrast normalization, multi-scale region enhancement to significantly improve the feature representation at different scales and sub-modalities. Besides, the contextual feature augmentation and saliency-based region extraction enhances the local feature description and guides the feature selection around the region of interest rather than systematic thus avoiding drawbacks that arise with the traditional preprocessing techniques. The integration with CASDN and detailed preprocessing considered in this work helps to apply robust segmentation for a variety of imaging types and fills the gaps of the existing research, providing a new framework for breast cancer detection and segmentation.

**Table 1:** Comparative analysis of existing segmentation methods for breast cancer detection.

Authors	Year	Methodology	Key Contributions	Limitations
Krishnakumar et al. [20]	2023	OTDEM-based segmentation with CLAHE, deep joint segmentation, and ensemble classification.	Enhanced image preprocessing with CLAHE; Multi-stage process including deep segmentation and feature extraction; Ensemble classifiers for robust results.	Complexity of multi-stage process; Requires significant computational resources; Ensemble models might overfit.
Lee et al. [21]	2020	Channel attention module with multiscale grid average pooling (MSGRAP) for ultrasound images.	Effective use of global and local spatial information for precise segmentation; Novel architecture for semantic segmentation.	Limited to ultrasound images; Requires extensive tuning of attention module; May not generalize well across different imaging modalities.
Mustafa et al. [22]	2014	Gradient Vector Flow (GVF) Snake technique for mammogram segmentation.	Effective for flexible boundary detection; Positive results in mammographic cancer detection.	Limited sample size; Sensitivity to initialization; May struggle with highly irregular boundaries.
Wu et al. [23]	2022	Two-stage framework leveraging pre- and post-contrast images with attention network.	Utilizes both pre- and post-contrast images for improved segmentation; Attention network refines ROI.	Dependence on high-quality contrast images; May be computationally intensive; Potential issues with varying contrast levels across datasets.
Zarei et al. [24]	2021	Hybrid Gaussian Mean Shift (GMS) and roulette wheel selection for infrared thermal images.	Hybrid approach combining GMS and roulette wheel selection; Effective for infrared thermal images segmentation.	Specific to thermal images; May not perform well with other imaging modalities; Clustering may be sensitive to parameter settings.
Samudrala et al. [25]	2024	Hybrid semantic segmentation using DenseNet-121 with Attention-based Pyramid Scene Parsing Network (Att-PSPnet).	Enhanced image contrast and feature extraction with attention mechanisms; Robust segmentation of high-dimensional features.	Potentially complex architecture; Requires substantial computational resources; May face challenges in real-time applications.
Zhou et al. [26]	2023	Faster Boundary-aware Transformer with boundary-wise attention block (BAB).	Improved local detail capture with boundary-wise attention; Efficient transformer decoder improvements.	Requires extensive training data; Potential overfitting with complex model architecture; May be challenging to implement.
Dong et al. [27]	2018	Reinforced Auto-Zoom Net (RAZN) for selective zooming in images.	Efficient zoom-in mechanism for balancing detail and noise; Robust to unbalanced labels and overfitting.	Limited to scenarios where zooming is applicable; Performance may vary with different datasets; Requires careful tuning of policy network.
Qin et al. [28]	2022	Two-stage TR-IMUnet model with improved ReLU for breast cancer lesion segmentation.	Enhanced segmentation with improved ReLU function; Effective for isolating tumor regions from non-relevant tissues.	Potential issues with model generalizability; May be sensitive to input data variations; Complexity in training process.
Qiao et al. [29]	2022	Marker-based watershed segmentation with local binary pattern and color auto-correlation features.	Effective for nuclei segmentation and feature extraction; Fusion of texture and color features for classification.	Requires accurate stain separation; Performance may be dependent on image quality and staining consistency; Complexity in feature extraction.
Xue Wang et al. [30]	2023	Diffusion-model-based instance segmentation (Diff-bcSeg).	Innovative use of diffusion models for denoising and segmentation; Effective for instance segmentation with minimal inductive bias.	Limited by the need for high-quality ground truth data; Computationally demanding; Potential challenges with noisy or ambiguous data.
Zeebaree et al. [31]	2019	Local pixel information-based neural network for ROI detection.	Simple yet effective approach for detecting ROI based on local pixel information; Easy to implement.	Limited to local pixel information; May not handle complex textures or boundaries effectively; Performance may vary with different image qualities.

## Method details

### System model

**Fig. 1:**
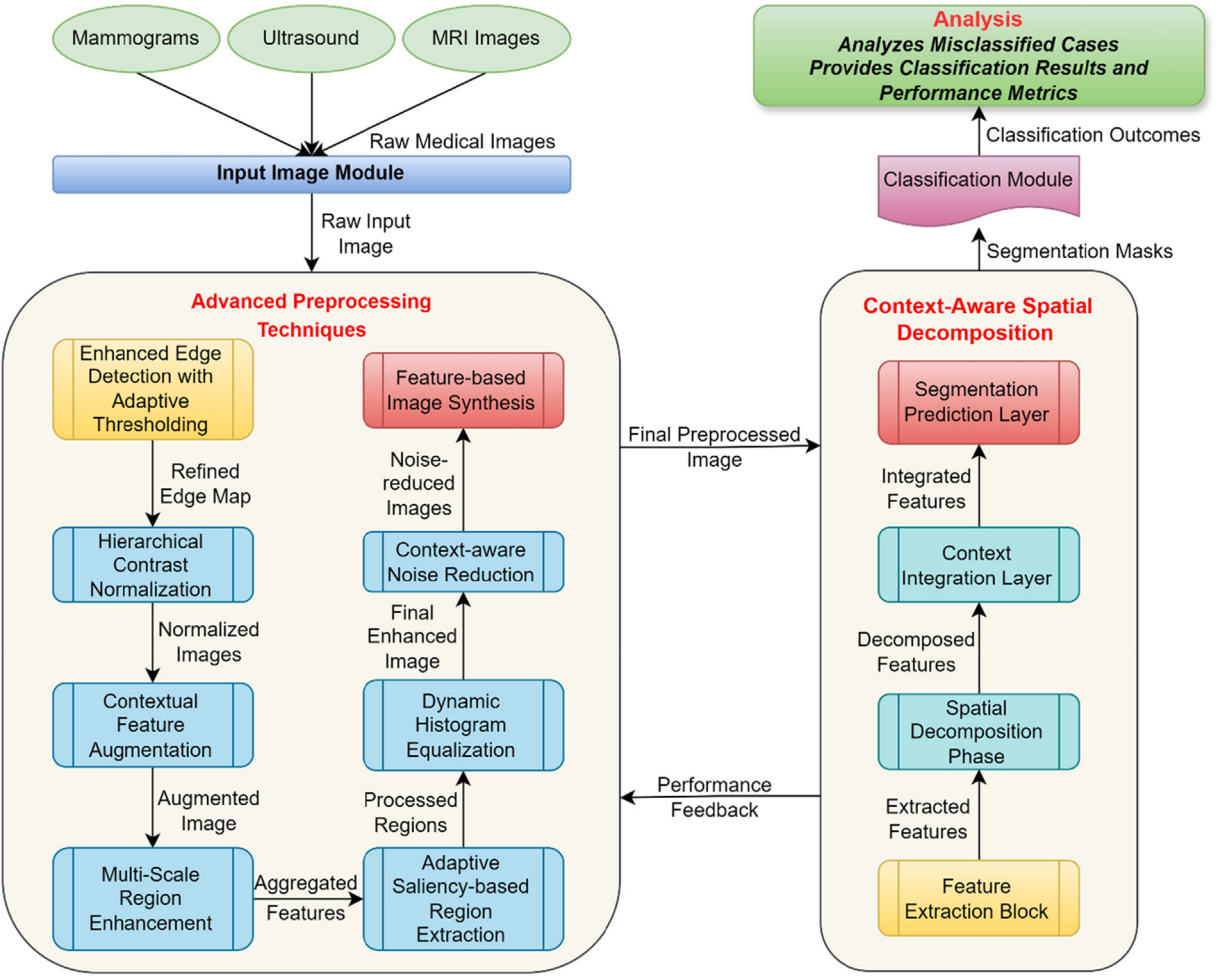
Proposed breast cancer segmentation framework.

The proposed work focuses on the segmentation of breast cancer images using Advanced Preprocessing Techniques with CASDN to overcome the challenges for high performance. It starts with the collection of raw medical images which can be in mammogram, ultrasound or MRI, etc. The above features constitute the raw images upon which the whole pipeline is built and are forwarded to the preprocessing module. Actually, the Advanced Preprocessing Module is a very important element in the enhancement of the image quality and the feature extraction as shown in Fig. 1. To start with, Enhanced Edge Detection with Adaptive Thresholding applies Sobel and Canny edge detectors in order to fine edge enhancement and adjust threshold values with regard to local gradient. This leads to correct representation of edge especially in the segmentation process which is very important. Subsequently, the Hierarchical Contrast Normalization operation takes place in the local and the global scenery for enabling visualization of features on multiple scales and eliminating problems of uneven lighting. Contextual Feature Augmentation follows to involve additional pixel information by computing key contextual maps using various Gaussian filters and combining with the earlier collected image data.

To strengthen the focus on numerous regions, the proposed framework has adopted Multi-Scale Region Enhancement where an image pyramid namely; features at different resolutions that include both fine details and global structures. After this, the technique Adaptive Saliency-based Region Extraction employ advanced saliency maps that are then enhanced using morphological operations. Last, Dynamic Histogram Equalization enhance a segment's brightness and contrast based on this segment and Context-Aware Noise Reduction maintains important details by reducing noise depending on local pixel variations. The received preprocessed image is then passed to the CASDN Framework, where context-aware spatial decomposition enables segmentation. The structured workflow provides better image representation quality resulting in better segmentation performance for predicting breast cancer.

### Motivation for advanced preprocessing techniques

Since conventional preprocessing techniques present the aforementioned challenges, it was underscored that superior solutions exist to meet such hurdles and enhance the quality and accuracy of breast cancer images. The motivation for developing and implementing advanced preprocessing techniques is driven by several factors:

i. **Enhanced Diagnostic Accuracy:** Some of the sophisticated preprocessing techniques are used to emphasize on improving the quality of the medical image thereby increasing of the accuracy of the diagnostic tools. Several of these techniques assist in minimizing on noise, artifacts, and other forms of image distortions that when eliminated enhance identification and or differentiation of cancer types.
ii. **Consistency Across Modalities:** As pointed out there is a group of methods, which are used for preprocessing data of different types of images, so there is demand for the methods, providing a similar reliable result. It is possible to develop complex methods that can be aimed at specific imaging modalities, as well as guarantee preprocessing efficiency no matter which technique is employed.
iii. **Improved Detection of Small Lesions:** Improved performance is one of the key objectives of advanced preprocessing because this approach helps to amplify small dark areas that may represent early-stage breast cancer. Thus, raising the contrast and resolution, one can rely on more precise signs in question to detect even such small pathologies.
iv. **Efficient Data Handling:** Due to advances in medical imaging, there is massive amount of data, which is also complex in nature. Deep preprocessing techniques that can operate on extended objects effectively are required to extract features from large sets that help to optimize the diagnostic process.
v. **Integration with Deep Learning:** Advanced preprocessing techniques are special in the sense that it can be tailored to complement machine learning and deep learning models. It should be noted that these techniques improve the diagnostic outcomes of the automated system and help in creating better input data sets for the construction of more precise ensuing predictive models.

### Overview of advanced preprocessing techniques

Augmented preprocessing methods for mammography include a set of techniques which cannot be classified as ordinary ones. These usually comprise of sophisticated forms of algorithms and models which are used to sharpen images or remove noise and artifacts or improve features. Some key areas of focus include:

i. **Noise Reduction:** To reduce the consequences of noise and artifacts on the medical images, wavelet denoising, bilateral filtering, and deep noise reduction estimators are used. These methods help in keeping as much detail away from the signal or image as possible, and getting rid of most interferences as well.
ii. **Contrast Enhancement:** Methods of contrast improvement are employed at the final stage; these methods include histogram equalization and adaptive contrast stretching. In this manner these techniques prove useful in distinguishing between malignant and benign tissues, by increasing the contrast.
iii. **Resolution Enhancement:** Techniques like super-resolution, and image upscaling, are used as methods for increasing the resolution of the medical images. Higher resolution helps to identify smaller nodules, which results in the overall diagnostic ability of the system.
iv. **Artifact Correction:** Correcting distortions and artifacts that may occur during image acquisition is another goal of advanced preprocessing techniques. To deal with problems like motion artifacts along with geometric distortions, methods like picture registration as well as correction algorithms are used.
v. **Integration with Machine Learning:** The combination with advanced preprocessing techniques also implies that the feature extraction is done more effectively. Thus, these techniques improve the assessment of machine learning algorithms as well as improve the diagnostics of diseases due to the availability of high-quality input data.

The integration of complex preprocessing techniques in the breast cancer images can be marked a revolution in the medical image analysis. Overcoming the shortcomings of the previously used approaches and concentrating on image improvement, noise elimination, and feature detection, these techniques improve breast cancer diagnosis outcomes. The desire to increase diagnostic accuracy and conformity between different imaging types, as well as facilitating data management and compatibility with the machine and deep learning models, justifies the motivation for the creation and implementation of such methods. With the emergence of steady progression in the field of medical imaging, preprocessing methods are expected to become the cornerstones in the early diagnosis of breast cancer to enhance the treatment results.

### Preprocessing steps for CASDN

The preprocessing pipeline of CASDN is carefully constructed to provide the best image for segmentation by CASDN. It starts with the input image which is the raw data to the entire preprocessing process. First, when using the Enhanced Edge Detection with Adaptive Thresholding, a Sobel edge detector identifies some minor edges, followed by a Canny edge detector that refines the edges more adaptively. The results are integrated or combined at the Edge Fusion Layer to generate an enhanced edge map [32]. After edge detection the pipeline uses Hierarchical Contrast Normalization as the next step. First, the image contrast at a local area or windows is corrected by Local Contrast Normalizer. This is done by the Global Contrast Normalizer which maintains the contrast in the whole picture to eliminate variation in illumination. The next level in Caffe is the Contextual Feature Augmentation which applies a Gaussian Filter to come up with alike images. In the Feature Fusion Layer, the smoothed images are added with the original image in order for the image enhancement through incorporated contextual information. Multi-scale Region Enhancement is then performed, the Image Pyramid Generator actually generates multiple scaled versions of the image. These are processed by the Feature Enhancer in which features at each scale of operation are modified. Then the Feature Aggregator will maintain these multi-scale features and compile them to create an overall enhanced image.

Next is Adaptive Saliency-based Region Extraction, where Saliency Map Generator produces maps targeting the regions of interest. These maps steer the Region Extractor and isolates the regions detailed and refines them with morphological operations. The Histogram Equalizer in Dynamic Histogram Equalization utilizes histograms of image segments computed by the Histogram Equalizer for controlling brightness and contrast of images so that all images are of equal quality. The last data preprocessing step is Context-aware Noise Reduction. The Noise Reduction Filter uses local noise reduction techniques to give a cleaner picture which is enhanced in Feature-based Image Synthesis. Here, the Feature Extractor captures feature descriptors from the resultant noise-reduced picture and Feature Synthesizer composes from these descriptors the final preprocessed picture. This structured flow allows the image to be thoroughly enhanced taking into account edge details, contrast, context features, multiple scale information, saliency, histograms and denoise. The end product is an ideal input for segmented analysis by the CASDN framework, with high quality worthy of utilization.

### Work flow

**Fig. 2:**
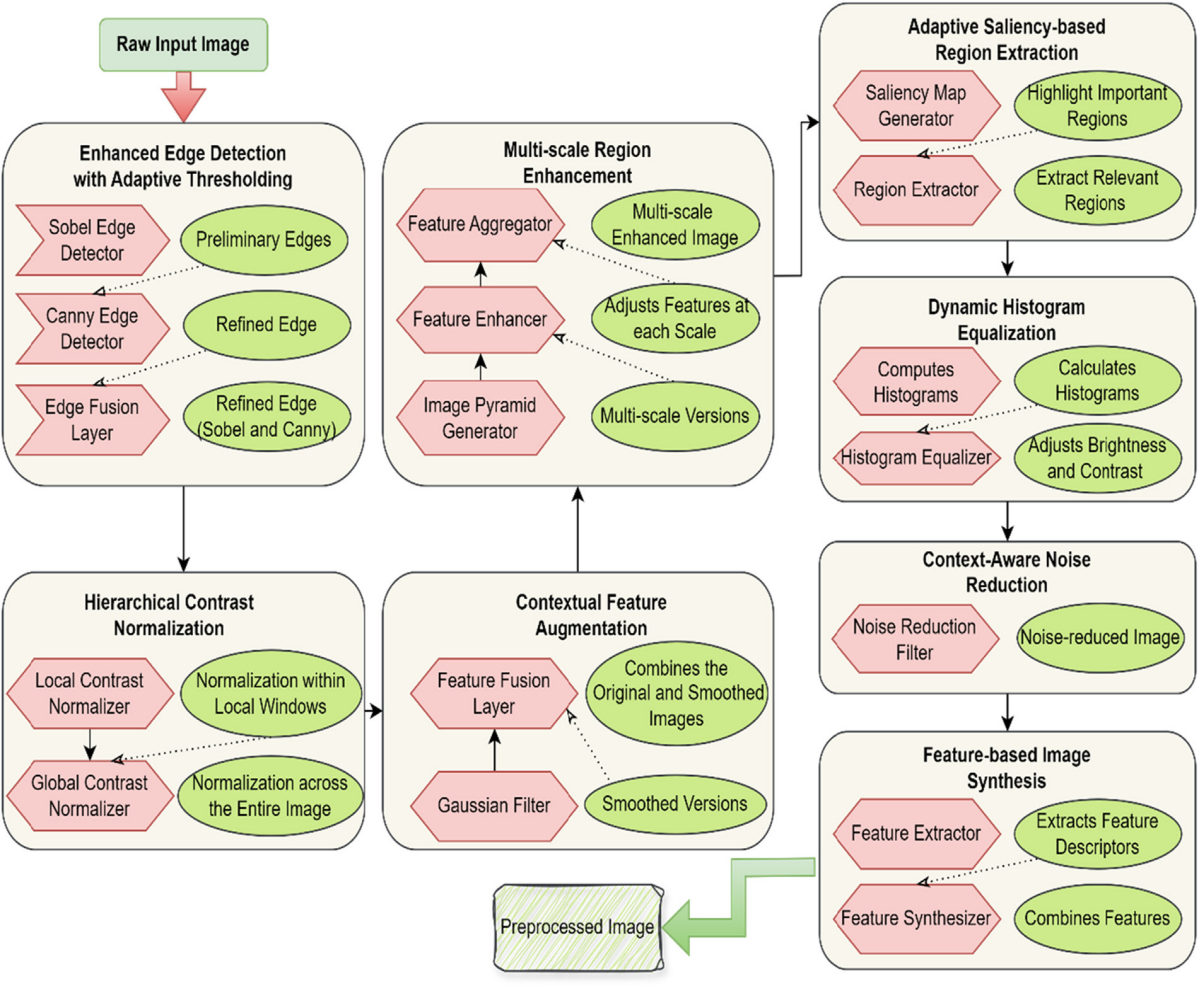
Preprocessing steps for CASDN.

***Enhanced Edge Detection with Adaptive Thresholding:*** Apply a hybrid edge detection method that combines Sobel and Canny edge detectors as shown in Fig. 2. This dual-layer approach enhances edge precision by adapting the threshold dynamically based on local image characteristics.

i. Use the Sobel operator to detect preliminary edges.
ii. Refine these edges using the Canny edge detector with a threshold dynamically adjusted by analyzing local gradient magnitude distributions.

***Hierarchical Contrast Normalization:*** Normalize image contrast hierarchically to enhance feature differentiation across varying scales.

i. Apply local contrast normalization using a sliding window approach.
ii. Normalize the contrast at different levels of image scales to ensure uniform feature representation and reduce variability caused by lighting conditions.

***Contextual Feature Augmentation:*** Integrate contextual information to enhance local features by analyzing surrounding pixel information.

i. Generate contextual maps by applying a Gaussian filter to the image to create smoothed versions.
ii. Combine these maps with the original image using a feature fusion approach that incorporates weighted pixel values based on their contextual relevance.

***Multi-Scale Region Enhancement:*** Enhance regions of interest at multiple scales to capture fine details and global structures.

i. Create a multi-scale image pyramid where each level represents different resolutions of the image.
ii. Apply feature enhancement operations (e.g., contrast adjustment, sharpening) at each scale, and then aggregate these enhanced features into a unified representation.

***Adaptive Saliency-based Region Extraction:*** Extract regions based on adaptive saliency maps to focus on important areas with high feature variance.

i. Generate saliency maps using an advanced saliency detection algorithm that adapts to varying image content.
ii. Extract regions of interest based on high saliency scores and refine these regions using morphological operations to remove noise and improve accuracy.

***Dynamic Histogram Equalization:*** Apply dynamic histogram equalization to adjust image brightness and contrast adaptively.

i. Compute histograms for different image segments.
ii. Apply equalization locally and globally to adjust pixel intensity distribution, enhancing contrast in areas of low visibility while preserving overall image details.

***Context-Aware Noise Reduction:*** Use context-aware noise reduction algorithms to remove noise while preserving important image features.

i. Apply a noise reduction filter that uses local context information to adaptively adjust filtering strength based on pixel variance and neighboring pixel information.

***Feature-based Image Synthesis:*** Synthesize new image features by combining information from original and enhanced images.

i. Extract feature descriptors from preprocessed images.
ii. Use a feature synthesis method to create a composite image that emphasizes key characteristics and enhances detail relevant to segmentation.

These preprocessing steps aim to improve the quality of input images for the CASDN by enhancing edges, normalizing contrast, integrating contextual information, and refining region extraction. Each step contributes to generating a more accurate and detailed representation of the image, facilitating better segmentation performance in the CASDN framework.

**Table:** 

**Algorithm for Advanced Preprocessing Techniques**

**Input:**
Raw breast cancer image I of size H×W
**Initialization:**
Set preprocessing parameters:
• **Edge Detection:** Sobel and Canny threshold values $T_{Sobel}$ and $T_{Canny}$
• **Contrast Normalization:** Sliding window size $W_{c}$
• **Contextual Augmentation:** Gaussian filter parameters $\sigma_{c}$
• **Multi-scale Pyramid Levels:** Number of levels $L_{pyr}$
• **Saliency Detection:** Saliency threshold $T_{saliency}$
• **Noise Reduction:** Filter strength $\lambda_{noise}$
• **Dynamic Histogram Equalization:** Segmentation bin size $B_{hist}$
**Preprocessing Steps:**
1. **Enhanced Edge Detection with Adaptive Thresholding:**
**Step 1:** Apply Sobel edge detection to obtain preliminary edges $E_{Sobel}$.
$E_{Sobel}=\;Sobel\left(I\right)$
**Step 2:** Refine edges using Canny edge detection with adaptive thresholding.
$E_{\left\{Canny\right\}}=\;Canny\left(I,\;T_{Canny}\right)$
**Step 3:** Combine edges using a weighted fusion approach.
$E_{Final}=\alpha_{edge}\cdot E_{Sobel}+\left(1-\alpha_{edge}\right)\cdot E_{Canny}\;$
2. **Hierarchical Contrast Normalization:**
**Step 4:** Apply local contrast normalization using a sliding window approach.
$I_{\left\{Norm\right\}}=\;Normalize\left(I,\;W_{c}\right)$
**Step 5:** Perform contrast normalization at multiple scales.
$I_{Final}=\;Normalize_{multi}-scale\left(I_{Norm},\;L_{pyr}\right)$
3. **Contextual Feature Augmentation:**
**Step 6:** Generate contextual maps by applying Gaussian smoothing.
$C_{map}=\;\;G_{\sigma c}*\;I_{Final}$
**Step 7:** Fuse contextual maps with the original image using feature fusion.
$I_{Augmented}=\beta \cdot I_{Final}+\left(1-\beta \right)\cdot C_{map}$
4. **Multi-Scale Region Enhancement:**
**Step 8:** Create a multi-scale image pyramid.
${\left\{I_{pyr}^{\left(l\right)}\right\}}_{l=1}^{L_{pyr}}=\;Pyramid\left(I_{Augmented},\;L_{pyr}\right)\;$
**Step 9:** Apply feature enhancement operations at each scale and aggregate features.
$I_{Enhanced}=\;\;Aggregate\;\left(Enhance_{multi}-scale\left(\left\{I_{pyr}^{\left(l\right)}\right\}\right)\right)$
5. **Adaptive Saliency-based Region Extraction:**
** Step 10:** Generate saliency maps to identify regions of interest.
$S_{map}=Saliency\left(I_{Enhanced},T_{saliency}\right)$
**Step 11:** Extract and refine regions based on saliency scores.
$R_{Extracted}=Morphological\;Refinement\left(S_{map}\right)$
6. **Dynamic Histogram Equalization:**
**Step 12:** Compute histograms for image segments.
$H_{segment}=Histogram\left(I_{Enhanced}\right)$
**Step 13:** Apply histogram equalization locally and globally.
$I_{Equalized}=Equalize\left(I_{Enhanced},H_{segment},B_{hist}\right)$
7. **Context-Aware Noise Reduction:**
**Step 14:** Apply noise reduction using a context-aware filter.
$I_{Noise\;Reduced}=Noise\;Reduction\left(I_{Equalized},\lambda_{noise}\right)$
8. **Feature-based Image Synthesis:**
**Step 15:** Extract feature descriptors from pre-processed images.
$F_{Descriptors}=ExtractFeatures\left(I_{Noise_{Reduced}}\right)$
**Step 16:** Synthesize a composite image emphasizing key characteristics.
$I_{Final\;Preprocessed}=Synthesize\left(F_{Descriptors},I_{Noise_{Reduced}}\right)$
**Output:**
Return the final preprocessed image $I_{Final\;Preprocessed}$​ as input to the CASDN framework.

### Context-Aware spatial decomposition network (CASDN)

**Fig. 3:**
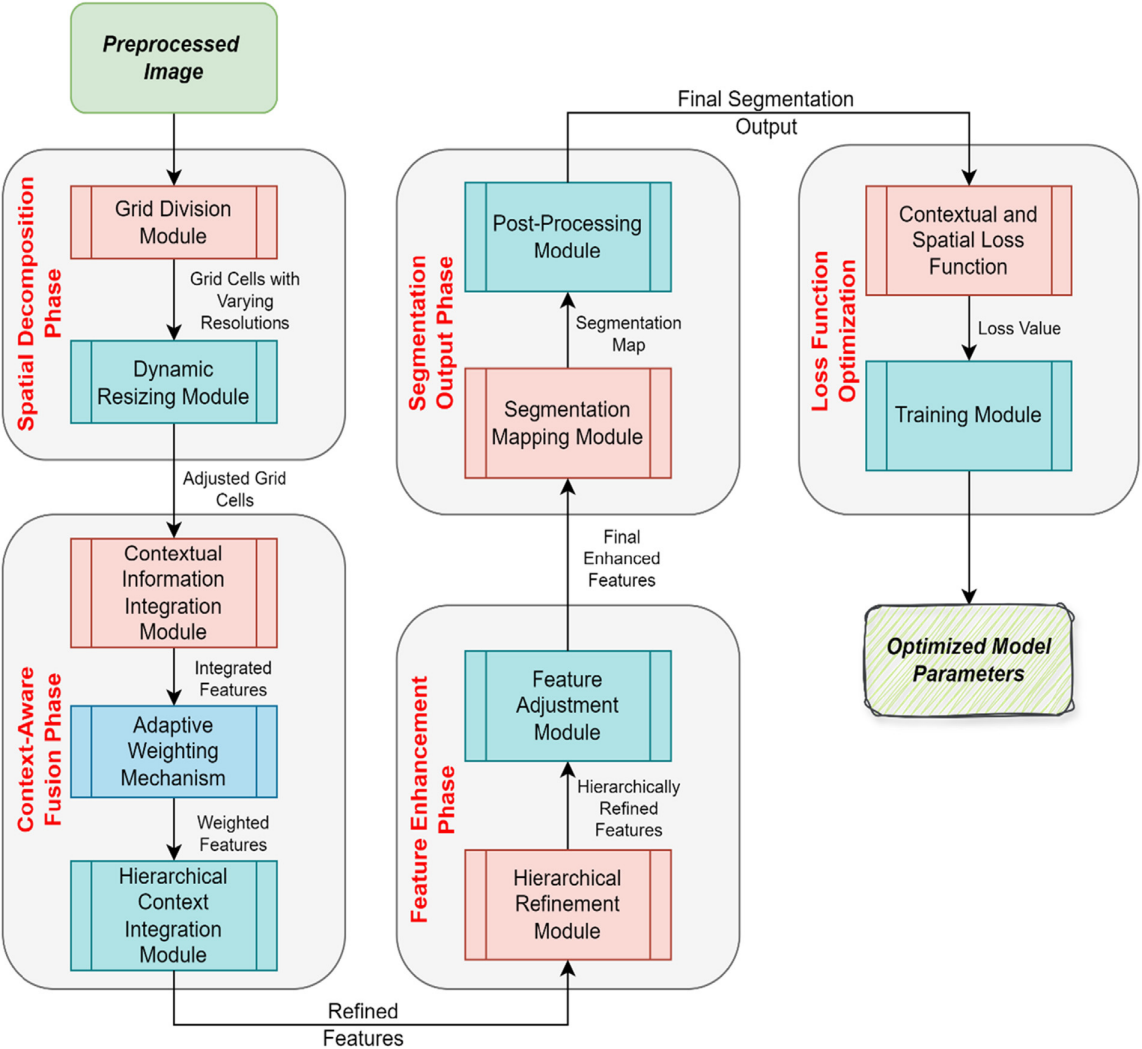
Context-aware spatial decomposition network (CASDN) framework.

The Context-Aware Spatial Decomposition Network (CASDN), proposes a novel approach for image segmentation that incorporates both the spatial decomposition and context integration as illustrated in Fig. 3. This algorithm can be initiated by approximating the image into a series of regions of different resolutions that enrich the investigation of spatial features. It is noteworthy that each of the grids receives independent processing with the ability to expand/reduce the analyzed region and adapt boundaries depending on the details considered and the general structural patterns as shown in Fig. 3. After the first level of decomposition, CASDN performs cross-grid cell fusion of information in a context-aware manner. This fusion layer uses adaptive weights for the number of features gradually to give the local features the priority over the spatial or human context in order to produce an adequate balance. The hierarchical context integration improves the work of the segmentation by combining at different levels the appearance of features taking into account the results of previous layers to achieve better features representation and segmentation results. To this end, CASDN employs a new loss function that integrates spatial similarity within a context to control the segmentation result at both the local level and by comparison with the global optimum. Not only does this approach increase accuracy, but it also enhances its performance depending on the image conditions. The proposed method is particularly useful in medical image analysis, remote sensing, and similar applications that require greater levels of accuracy in segmentation.

#### Phases of CASDN

**Spatial Decomposition Phase**

***Grid Division:*** The preprocessed image is divided into a grid of regions with varying resolutions. This decomposition allows the network to analyze different spatial characteristics in detail.

***Fine-Scale Cells:*** Capture fine details from high-resolution regions.

***Coarse-Scale Cells:*** Capture broader structural information from low-resolution regions.

***Dynamic Resizing:*** Each grid cell is dynamically resized and adjusted at the boundaries to ensure an accurate representation of features across different scales.

**Context-Aware Fusion Phase**

***Contextual Information Integration:*** Features from different grid cells are integrated using a context-aware fusion mechanism.

***Adaptive Weighting:*** Features are prioritized based on their spatial and contextual relevance. This ensures that both local details and global context are effectively combined.

***Hierarchical Context Integration:*** Features are processed at multiple levels, with each level building on previous layers to enhance representation and segmentation accuracy.

***Fusion Layer:*** Combines information across different grid cells to produce a comprehensive feature map that reflects both local and global image characteristics.

**Feature Enhancement Phase**

***Hierarchical Refinement:*** Refines features at multiple hierarchical levels to improve segmentation accuracy.

***Iterative Processing:*** Each hierarchical level processes the features from previous levels to iteratively build a more refined representation.

***Feature Adjustment:*** Features are adjusted based on their relevance and contribution to the final segmentation output.

**Segmentation Output Phase**

***Segmentation Mapping:*** The enhanced features are used to generate the final segmentation map.

***Prediction Layer:*** Uses the refined features to classify each pixel or region into different categories.

***Boundary Detection:*** Ensures accurate delineation of boundaries between different segments.

***Post-Processing:*** Applies any necessary post-processing steps to improve the quality of the segmentation output. Smoothing reduces any artifacts or irregularities in the segmentation map. Refinement further refines the segmentation boundaries and accuracy.

**Loss Function Optimization**

***Contextual and Spatial Loss:*** Utilizes a novel loss function that balances spatial coherence with contextual relevance.

***Spatial Coherence:*** Ensures that neighboring regions are consistently segmented.

***Contextual Relevance:*** Adjusts for global context to improve overall segmentation performance.

***Training Process:*** Optimizes the CASDN parameters to minimize the loss function, thereby improving segmentation accuracy.

This phased approach ensures that CASDN effectively leverages the preprocessed image data to achieve accurate and context-aware segmentation results.

#### CASDN algorithm

**Input:**

  Preprocessed Image $I_{Final\;Preprocessed}$

**Initialization:**

  Set the number of grid cells $N_{cells}$​.

  Initialize context-aware fusion parameters $\alpha_{context}$ and $\beta_{context}$​.

  Define hierarchical integration levels $L_{context}$.

  Set loss function parameters $\lambda_{loss}$.

**Segmentation Steps:**

**(a). Spatial Decomposition:**

**Step 1:** Decompose the input image $I_{Final\;Preprocessed}$​ into a grid of regions. Each region $R_{i}$ is extracted with varying resolutions to capture detailed spatial characteristics.

$${\left\{R_{i}\right\}}_{i=1}^{N_{cells}}=\;Decompose\left(I_{Final\;Preprocessed},N_{cells}\right)$$

**Step 2:** Process each grid cell $R_{i}$​ individually to extract relevant features.

$$R_{i}^{\left\{processed\right\}}=\;Process\left(R_{i}\right)$$

**(b). Context-Aware Fusion:**

**Step 3:** Fuse features from different grid cells using a context-aware mechanism. This involves calculating a weighted sum of features based on spatial and contextual relevance.

$$F_{fused}=\;\sum_{i=1}^{N_{cells}}w_{i}.R_{i}^{\text{processed}}$$

where $w_{i}$ is the adaptive weight for grid cell $R_{i}$ ​, computed using:

$$w_{i}=\frac{\text{exp}\left(\alpha_{context}.\;Relevance(R_{i}\right))}{\sum_{i=1}^{N_{cells}}\text{exp}\left(\alpha_{context}.\;Relevance(R_{i}\right))}$$

**Step 4:** Apply adaptive weighting to prioritize features based on their relevance.

$$F_{adaptive}=Adaptive\;Weighting\left(F_{fused},\;\beta_{context}\right)$$

**(c). Hierarchical Context Integration:**

**Step 5:** Integrate features at multiple hierarchical levels to build a comprehensive representation.
  $$F_{hierarchical}^{l}=\;Hierarchical\;Integration\left(\left(F_{adaptive},\;L_{context}^{\left(l\right)}\right)\right)$$

where l denotes the hierarchical level and $L_{context}^{\left(l\right)}$​ represents the contextual parameters at level l.

**Step 6:** Perform iterative refinement on the hierarchical features to enhance representation accuracy.

$$F_{refined}=F_{hierarchical}+\beta_{refine}\cdot Update\left(F_{hierarchical}\right)$$

where $\beta_{refine}\;$is a refinement parameter and $Update(F_{hierarchical})$ represents an iterative adjustment function that enhances the feature representation based on hierarchical features.

**(d). Loss Function Optimization:**

**Step 7:** Compute the loss function that balances spatial coherence and contextual relevance.

$$L_{CASDN}=\lambda_{loss}\cdot \left[\alpha_{spatial}\cdot Spatial\;Loss\left(F_{refined}\right)+\alpha_{context}\cdot Context\;Loss\left(F_{refined}\right)\right]$$

where $\alpha_{spatial}$​ and $\alpha_{context}$ are weighting factors for spatial and contextual losses respectively, and $Spatial\;Loss(F_{refined})$ and $Context\;Loss(F_{refined})\;$are the spatial and contextual loss functions applied to the refined features.

**Step 8:** Optimize model parameters based on the computed loss.

$$Optimize\left(L_{CASDN}\right)$$

**Output:**

Return the segmented output $F_{refined}$​.

**Method validation**

## Results and discussion

For CASDN performance evaluation, we employ an Intel Core i7–10700 K CPU that runs in a stable computing environment with an NVIDIA GeForce RTX 3080 Graphics Processing Unit and 16 GB RAM. The CASDN was compared against numerous conventional segmentation methods, including DeepLabV3+, Otsu's Thresholding, Region Growing, Watershed algorithm, and U-Net. For the performance evaluation, the experiments were conducted using two publicly available datasets: The DMR-IR (Frontal Thermal Images) had a resolution of 640 * 480 pixels and the CBIS-DDSM (Mammogram) dataset had a resolution of 1024 * 1024 pixels. These datasets which are commonly used in medical imaging studies contain a large amount of breast cancer images in various modalities, which is sufficient for evaluating the effectiveness of preprocessing for image classification and performance of the whole system.

### Comparison and analysis of learning and classification models

In the case of the classification model performance, the changes that take place before and after the application of Advanced Preprocessing Techniques show that this method has a positive impact on the several parameters. This enhancement particularly highlights the effectiveness of the proposed preprocessing technique in the image data screening process, and the improvement in classification results. The improvement of the performance measurements for CNNs was achieved by the sale of sophisticated preprocessing algorithms. The initial CNN model has a testing accuracy of 78.5 % for the given data set upon the pre-processing phase while the precision, recall and F1 score recorded were, 76.2 %, 80.1 and 78.0 % respectively. When the data was preprocessed, the accuracy was improved up to 85.3 % and the precision up to 83.9 %, the recall up to 86.0 %, and F1 score up to 84.9 %. The AUC-ROC was also improved from 0.82 to 0.90 as presented in Table 2. This improvement proves that the preprocessing techniques applied enhanced the model capacity in correctly classifying images of breast cancer resulting in reduced false positive and more precise identification of positive cases.

**Table 2:** Performance evaluation before advanced preprocessing.

Model	Accuracy	Precision	Recall	F1 Score	AUC-ROC
Convolutional Neural Networks	78.50	76.20	80.10	78.00	0.82
Recurrent Neural Networks	71.40	69.80	72.00	70.90	0.76
K-Nearest Neighbors	66.30	65.10	67.50	66.30	0.72
Generative Adversarial Networks	72.80	70.50	74.20	72.30	0.74
Long Short-Term Memory Networks	68.90	67.30	70.10	68.70	0.70
Support Vector Machines	75.10	73.60	76.50	74.90	0.79
Random Forest	74.20	72.70	76.00	74.20	0.77

**Fig. 4:**
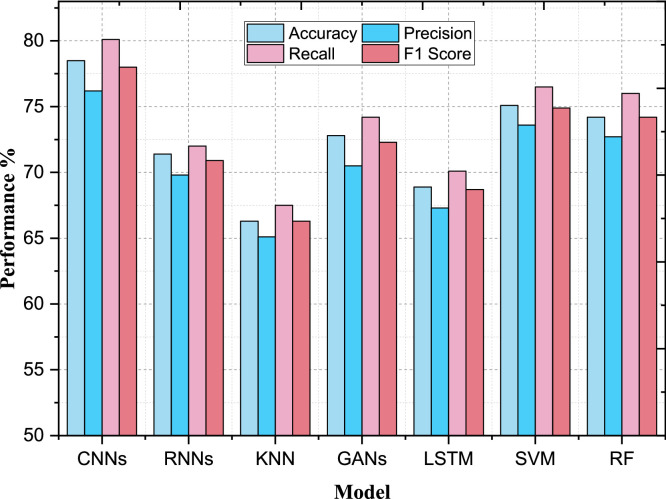
Performance evaluation before advanced preprocessing.

**Fig. 5:**
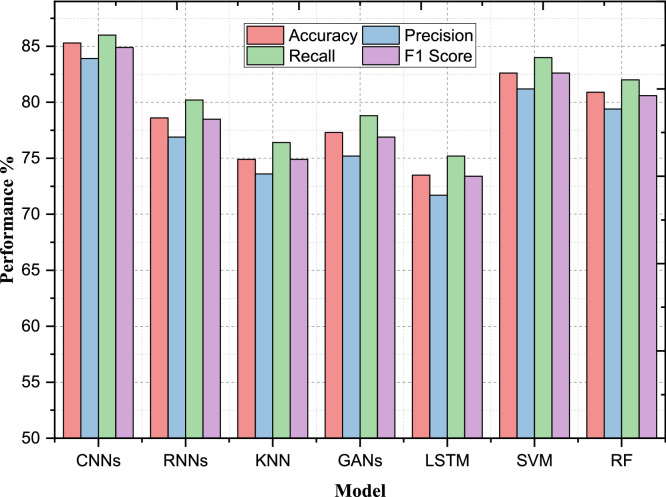
Performance evaluation after advanced preprocessing.

Likewise, there has been a considerable development showcased by SVMs with the integration of the ideal enhanced preprocessing techniques. Accuracy increased to 82.6 % from 75.1 % for the dataset, precision improved from 73.6 % to 81.2 % and recall from 76.5 % to 84.0 % for the dataset. From the Fig. 4, Fig. 5, it was found that the F1 score was improved almost to 82.6 % from 74.9 % and for AUC-ROC from 0.79 to 0.87. These changes state that in addition to improving on the accuracy of the model, the preprocessing techniques also solve the problems of true positives as well as minimizing the false positives hence making the SVM model better.

The KNN model also got the advantage with the advanced preprocessing done on the data. In the non-preprocessed data, KNN had an accuracy of 66.3 %, precision of 65.10 %, recall of 67.5 and F1 score of 66.3 %. After utilizing AIP, accuracy increased to 74.9 %, precision to 73.6 %, recall to 76.4 % and F1 score to 74.9 %. There was an improvement of the AUC-ROC, from 0.72 to 0.80. These improvements indicate that preprocessing predicated somewhere near the KNN model's performance improvement which bring out the clarity of its classification level plus the discrimination of positive and negative examples. The ability to apply enhanced preprocessing techniques proved successful in enhancing and achieving the optimal results of multiple classification models. In addition to the structural enhancement of image data, the preprocessing increased both the accuracy, precision, recall, F1 score, and area under the curve–receiver operating characteristic (AUC-ROC) of the models. This goes to prove how important preprocessing is in order to attain more improved reliability and accuracy of the classification when it comes to breast cancer image analysis. A comparison of CNNs and SVMs before and after the application of the advanced preprocessing techniques has shown significant enhancements. On the other hand, the CNN model accuracy was 78.5 %, it had a precision of 76.2 %, while SVM model had an accuracy of 75.1 % and had a precision of 73.6 % before preprocessing. This suggested that CNNs had a natural predisposition for processing the raw image data, while both models were confined in their general performance by the lack of complex preprocessing.

The result of the CNN model is greatly improved after applying the advanced preprocessing techniques as presented in Table 3, where the accuracy reached 85.3 %, and precision was 83.9 %. This enhancement shows that preprocessing was indeed beneficial in improving feature extraction, and in minimizing noise which helped the CNN to learn a clear distinction between malignant and benign tissues. The SVM model also foreseen a boost from preprocessing with accuracy going up to 82.6 %, precision up to 81.2 %. Subsequently, the relative improvement of the CNN model was considerably higher. This proves that apart from enhancing the CNN's ability to process data, the preprocessing techniques also offered enhancements to normal classifiers such as the SVM. The tables of accuracy presented below show how much better GANs as well as RF perform after the data has been preprocessed and PCA performed. To start with, the feedback analysis of the initial GAN model had an F1 Score of 72.3 % and AUC-ROC of 0.74 as compared to the RF model with low F1 Score of 74.2 % and AUC-ROC of 0.77 is represented in Fig. 5. These figures indicate the fact that both these models struggled with unprocessed image data.

**Table 3:** Performance evaluation after advanced preprocessing.

Model	Accuracy	Precision	Recall	F1 Score	AUC-ROC
Convolutional Neural Networks	85.3	83.9	86	84.9	0.90
Recurrent Neural Networks	78.6	76.9	80.2	78.5	0.84
K-Nearest Neighbors	74.9	73.6	76.4	74.9	0.80
Generative Adversarial Networks	77.3	75.2	78.8	76.9	0.81
Long Short-Term Memory Networks	73.5	71.7	75.2	73.4	0.77
Support Vector Machines	82.6	81.2	84	82.6	0.87
Random Forest	80.9	79.4	82	80.6	0.84

After the post preprocessing the F1 score of the model of GAN is 76.9 % and the AUC-ROC value of the GAN is 0.81. This large improvement further demonstrated that preprocessing benefited the GAN model when generating better and more reliable segmentations from high-quality input data. Likewise, the F1 Score of the RF model was 80.6 % and the AUC- ROC was 0.84 for the current experiment. This implies that the advanced preprocessing provided improvements to the performance of the segmentation to both models through enhancements in feature maps. All these scenarios support that the proposed advanced preprocessing methods can improve the classification models by improving and standardizing input data, improving feature extraction, and improving model accuracy and credibility. High-resolution images are largely given in this dataset, which is necessary for the assessment of the performance and inapplicability of segmentation techniques. To further illustrate the analysis performed in the previous section in relation to the performance of CASDN in classifying breast cancer areas, a benchmark was done with respect to Hausdorff Distance, Boundary Accuracy, Intersection of Union (IoU), and Dice Co-efficient which is illustrates in Table 4.

**Table 4:** Segmentation performance comparison table.

Method	Dice Coefficient	IoU	Boundary Accuracy	Hausdorff Distance
Otsu's Thresholding	0.72	0.64	0.68	15.2
Region Growing	0.76	0.70	0.73	12.5
Watershed Algorithm	0.78	0.72	0.75	10.9
U-Net	0.84	0.80	0.82	8.6
DeepLabV3+	0.85	0.81	0.83	7.4
CASDN (Proposed)	0.89	0.85	0.87	5.2

#### Dice coefficient (Dice similarity coefficient, DSC)

The Dice Coefficient measures the intersection between two sets, which, in the background of image segmentation, are stereotypically the ground truth and the predicted segmentation mask. It is a normally used metric to assess the performance of segmentation algorithms.

$$Dice\;Coefficient\;\left(DSC\right)=\frac{2|A\cap B|\;}{|A|+|B|}$$

Where, ∣A∩B∣ is the number of pixels that are common in both the ground truth (A) and the predicted mask (B). ∣A∣ is the total number of pixels in the ground truth mask. ∣B∣ is the total number of pixels in the predicted mask. DSC ranges from 0 to 1, where 1 indicates seamless overlap between the ground truth and projected segmentation, and 0 indicates no overlap.

#### Intersection over union (IoU)

The Intersection over Union, also recognized as the Jaccard Index, measures the ratio of the intersection to the union of the ground truth and predicted masks. It is alternative common metric for evaluating segmentation performance.

$$\text{IoU}=\;\frac{|A\cap B|}{|A\cup B|}$$

Where, ∣A∩B∣is the number of pixels that are common to both the ground truth (A) and the predicted mask (B). ∣A∪B∣is the number of pixels in the union of the ground truth and predicted masks. IoU ranges from 0 to 1, with 1 representing a perfect match and 0 indicating no overlap.

#### Boundary accuracy

Boundary Accuracy measures the quality of boundary detection in segmentation. It assesses how well the predicted segmentation boundaries bring into line with the ground truth boundaries.

$$Boundary\;Accuracy=\frac{\text{Number}\;\text{of}\;\text{Correct}\;\text{Boundary}\;\text{Pixels}}{\text{Total}\;\text{Number}\;\text{of}\;\text{Boundary}\;\text{Pixels}}$$

Where, the number of correct boundary pixels is the number of boundary pixels in the predicted mask that match the boundary pixels in the ground truth mask. The total number of boundary pixels is the number of boundary pixels in the ground truth mask. Boundary Accuracy is articulated as a percentage and reflects how precisely the segmentation boundaries have been detected.

#### Hausdorff distance

The Hausdorff Distance measures the maximum distance between the boundary points of the ground truth and the projected segmentation. It is used to quantify the spatial discrepancy between the two boundaries.

$$\text{Hausdorff}\;\text{Distance}=\text{ma}x_{x\in A}\;\text{mi}n_{y\in B}∥x-y∥,\text{ma}x_{y\in B}\text{mi}n_{x\in A}∥x-y∥)$$

Where, A and B are boundary points of ground truth and predicted segmentation respectively, and ∥x-y ∥ is the Euclidean distance between the points x and y. Hausdorff Distance offers the assessment of the upper-bound boundary error. Thus, a smaller Hausdorff Distance means that boundaries of the actually predicted segmentation are nearer to the actual boundaries. It can be even more influential by outliers or significant differences from the mean. Each of these measurements contribute to the final evaluation of the segmentation quality and gives an account of the overall matching and the localization of the found boundaries. Below is a table of discrepancy that involves distinct sorts of segmentation techniques; here also representing the performance analysis of Context-Aware Spatial Decomposition Network (CASDN) against some existing strategies. The evaluation methods we employ are Dice Coefficient, Intersection over Union (IoU), Boundary Accuracy, Hausdorff Distance.

**Fig. 6:**
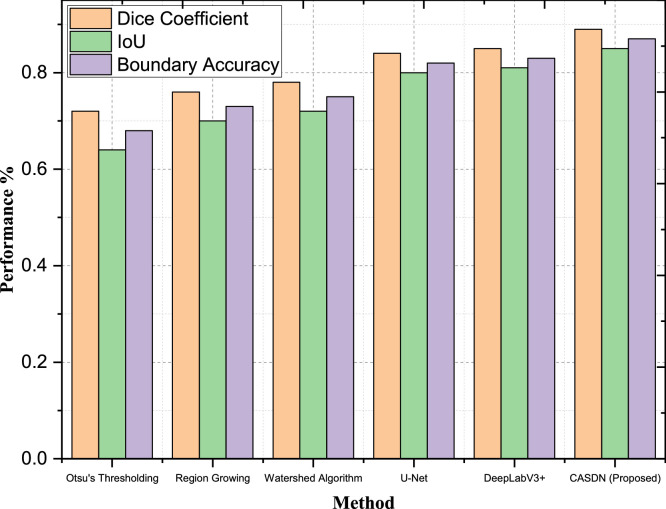
Segmentation performance - dice coefficient, intersection over union (IoU), and boundary accuracy.

In assessing the performance of the proposed Context-Aware Spatial Decomposition Network (CASDN), several scenarios demonstrate the applicability of the proposed approach over traditional segmentation methods. Firstly, CASDN obtains 0.89 Dice Coefficient which is significantly higher than traditional approaches like Otsu's Thresholding (0.72) and Region Growing (0.76) in addition to the advanced methods such as U-Net (0.84) & DeepLabV3+ (0.85) as described in Fig. 6. This superior Dice Coefficient shows that the proposed CASDN has better overlap degree with the ground truth segmentation than the other five methods for accurately detecting the details of breast cancer regions.

Additionally, the IoU of CASDN is increased to 0.85, which is significantly higher than the methods mentioned above. For example, IoU of U-Net is 0.80 while DeepLabV3+ has an IoU of 0.81. The higher IoU realized by CASDN tends to support these contentions, in that they demonstrate that the network can indeed well outline the cancer regions from the remainder of the tissue volume and map the segments as closely as possible to the actual regions of interest. Furthermore, the CASDN has a high Boundary Accuracy with a mean score of 0.87, which is higher than for other methods, such as Watershed Algorithm (0.75), and DeepLabV3+ (0.83). These features of improved Boundary Accuracy gave testimony to CASDN's ability to delineate with great precision the boundaries of tumorous tissues fundamental to diagnostic assessment and therapeutic planning.

**Fig. 7:**
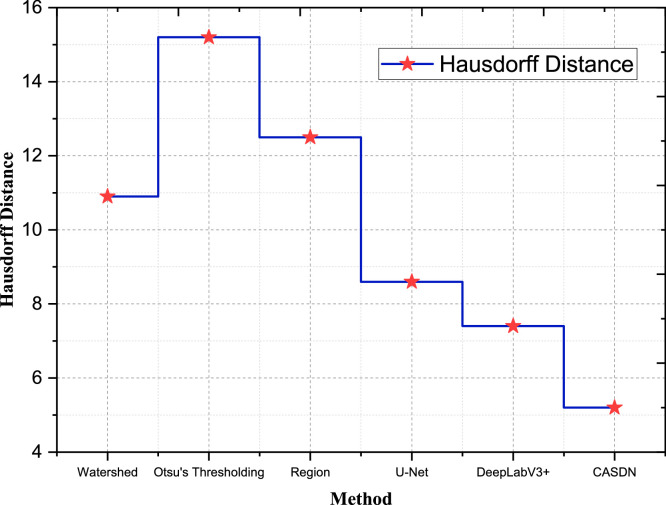
Segmentation performance - Hausdorff distance.

Finally, we calculate the CASDN's Hausdorff Distance 5.2 of the best performance compared to the other methods. For instance, Watershed Algorithm indicates the Hausdorff Distance of 10.9, and DeepLabV3+ is 7.4 according to Fig. 7. A lower Hausdorff Distance obtained by the proposed CASDN also indicates its ability to provide better segmentation accuracy in terms of minimizing the largest distance between layer boundary predictions and actual layer boundaries.

### Evaluation metrics for segmentation and structural accuracy

To ensure the robustness and clinical applicability of the proposed framework, we employed three more advanced evaluation metrics: Our evaluations depend on Dice Similarity Coefficient (DSC), Structural Similarity Index Measure (SSIM), and Proportion of Correct Patches (PCP). These metrics help assess how well the predictions match intended results for both their structure and internal regions to produce correct breast cancer diagnosis. The Dice Similarity Coefficient (DSC) helps us validate our results against ground truth data because it shows how well our predicted tumor edges match real tumor edges. Localizing malignant tissue areas accurately helps doctors make better medical choices in imaging data analysis. Robust segmentation results appear when DSC values stay high which helps avoid both incorrect positive and negative detections. The Structural Similarity Index Measure (SSIM) uses this method to measure image structure quality by matching luminance, contrast, and structural elements to ground truth data. Medical imaging professionals depend on SSIM to protect the proper structures of tissue and anatomy during segmentation before clinical analysis. The Proportion of Correct Patches measures how well localized prediction areas match their correct segments in an image. This measurement demonstrates that the method correctly detects cancer areas in multiple medical scanner systems and patient cases.

**Fig. 8:**
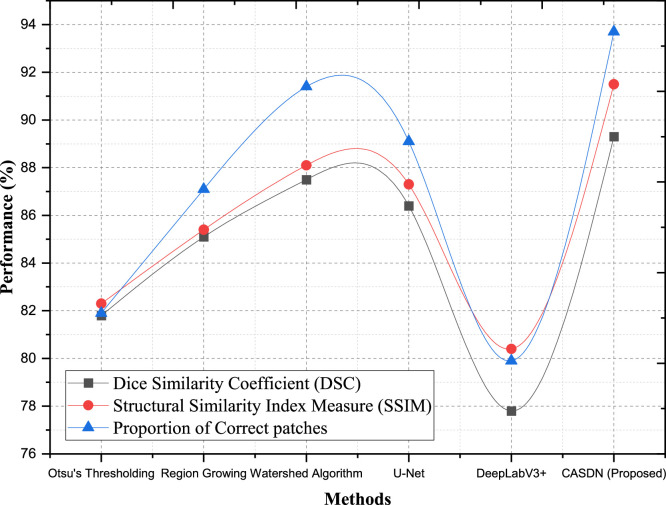
Segmentation and structural accuracy for dataset −1 (DMR-IR).

The Context-Aware Spatial Decomposition Network (CASDN) showed better performance than other segmentation approaches for Dataset 1 (DMR-IR) as shown in Table 5. Our proposed Context-Aware Spatial Decomposition Network (CASDN) demonstrates high robustness and precise segmentations when measured against Dice Similarity Coefficient (DSC), Structural Similarity Index Measure (SSIM), and Proportion of Correct Patches (PCP). CASDN produced an 89.3 % Dice Similarity Coefficient result that stood above all other number analysis methods. Our CASDN method shows 2.1 % better results than Watershed Algorithm's 87.5 % accuracy making it the top performer as shown in Fig. 8. CASDN achieves improved accuracy when detecting tumor regions because it matches well with the ground truth measurement. CASDN outperforms both Watershed and U-Net by delivering 91.5 % SSIM which represents 3.4 % and 4.2 % superior performance respectively. The metric proves CASDN's power to keep the breast cancer image structures intact and exact. In terms of the Proportion of Correct Patches (PCP), CASDN generates accurate segmentations at 93.7 % compared to Watershed Algorithm 91.4 %, the next best method, and shows an advantage of 4.6 % over U-Net's results. The model shows strong performance at identifying specific portions of tissue samples which enables dependable medical result evaluation. CASDN delivers better segmentation results according to all measurement criteria especially with 5.6 % superior PCP performance against region growing and 11.5 % better SSIM results compared to DeepLabV3+. Our results confirm that this framework can adapt to and precisely analyze breast cancer images and boost diagnostic workflow effectiveness.

**Table 5:** Comparison of segmentation and structural accuracy.

Datasets	Methods	Dice Similarity Coefficient (DSC)	Structural Similarity Index Measure (SSIM)	Proportion of correct patches
Dataset 1 (DMR-IR)	Otsu's Thresholding	81.8	82.3	81.9
	Region Growing	85.1	85.4	87.1
	Watershed Algorithm	87.5	88.1	91.4
	U-Net	86.4	87.3	89.1
	DeepLabV3+	77.8	80.4	79.9
	CASDN (Proposed)	89.3	91.5	93.7

Dataset 2 (CBIS-DDSM)	Otsu's Thresholding	83.5	84.3	85.6
	Region Growing	84.7	85.3	87.3
	Watershed Algorithm	89.3	88.6	91.6
	U-Net	87.9	88.1	90.3
	DeepLabV3+	79.4	82.7	80.3
	CASDN (Proposed)	91.3	90.8	95.4

**Fig. 9:**
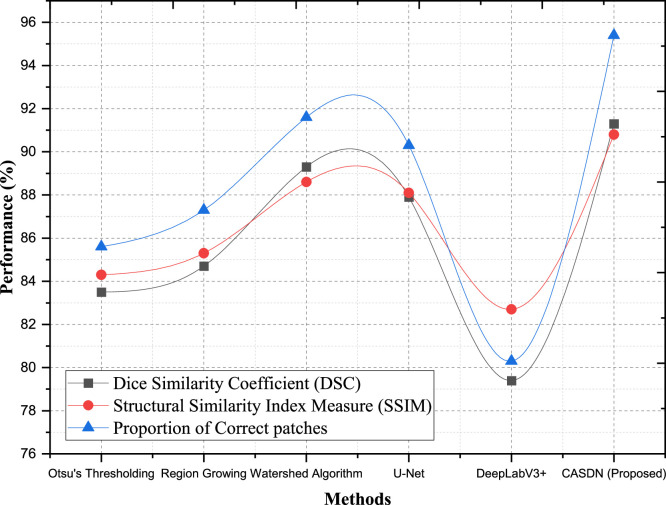
Segmentation and structural accuracy for dataset −2 (CBIS-DDSM).

Our Context-Aware Spatial Decomposition Network (CASDN) achieves impressive results on Dataset 2 (CBIS-DDSM) as shown in Table 5. The Dice Similarity Coefficient (DSC) measurement for CASDN records 91.3 % which comes 2 % higher than Watershed Algorithm (89.3 %) and 3.4 % higher than U-Net (87.9 %). CASDN demonstrates better performance in matching actual tumor boundaries because its enhanced ability leads to more accurate tumor identification. Using SSIM measurements CASDN shows 90.8 % score that outperforms both the Watershed Algorithm at 88.6 % and U-Net at 88.1 % as shown in Fig. 9. CASDN demonstrates reliable performance by capturing important breast cancer image structures needed for diagnostic precision. CASDN produces tumor region delineations whose accuracy is 95.4 % which stands 3.8 % better than Watershed being 91.6 % and 5.1 % higher than U-Net at 90.3 %. CASDN shows better tumor detection results in medical images than other methods because this strategy detects tumor areas more accurately throughout its processes. Our proposed method CASDN delivers better performance than Otsu Thresholding and Region Growing which results in a 12 % PCP increase and 7.5 % SSIM gain because it handles medical image complexities well. CASDN classification technology records superior results than DeepLabV3+ in both performance (11.9 %) and quality indexes (8.1 % for SSIM and 15.1 % for PCP). It demonstrates its effectiveness against various image challenges. Our framework demonstrates its accuracy to produce clinically appropriate segmentations through multiple testing methods. Our method demonstrates reliable performance across different breast cancer diagnostic settings in actual clinical practice.

### Limitations

The incorporation of Advanced Preprocessing Techniques and the CASDN proposed in this article exhibits promising results in breast cancer image segmentation along with certain enhancement. The first limitation is concerned with the preprocessing pipeline and CASDN where both approaches provide high accuracy and robustness, yet may be computationally intensive, and thus may be less ideal for real-time or low-cost application. Finally, frameworks such as the proposed one deliver outstanding performance across various imaging modalities; yet, replicating and validating the method on more extensive, heterogonous databases may also be beneficial. Future research could therefore, incorporate the use of explainability AI techniques to enhance decision making and which is very important in the medical field. Frontiers to the existing framework could be found in automating the hyperparameter tuning and the integration of adaptive learning mechanisms to the workflow. Furthermore, the generalization of the framework to other important MI tasks including but not limited to multi-class tumor classification, or analysis of disease progression over time is an enticing avenue of further exploration. These directions make sure the framework stays as an active and adaptive solution to the problem addressing medical image analysis.

## Conclusion

Breast cancer is a significant public health problem to this date and as such the need to develop new techniques for a better diagnosis still exists. In this work, a novel method is proposed to segment breast cancer images based on Advanced Image Preprocessing Techniques and a newly introduced Context-Aware Spatial Decomposition Network (CASDN). The proposed methodology offers novelty for deep learning methods by addressing the issues of image quality improvement, integration of contextual information and segmentation optimization. The developed preprocessing pipeline enhances image quality and feature representation at a very high level. Measures like hierarchical contrast normalization, adaptive edge detection, and multi-scale region enhancement cope with problems like inhomogeneous brightness, low contrast and noise. These enhancements help to ensure that the input images themselves are as good as possible for segmentation, thereby allowing CASDN to realise these advances. The CASDN framework successfully uses spatial decompositions and contextual integration; relevant tumor margins are well defined while achieving stable image processing performance under various conditions. Cross-sectional comparisons support the benefits of our approach. The proposed CASDN achieves a Dice coefficient of 0.89, Intersection over Union (IoU) of 0.85, boundary accuracy of 0.87, and a Hausdorff distance of 5.2, outperforming state-of-the-art methods such as DeepLabV3+. Moreover, the classification outcomes using preprocessing reveal that there are substantial enhancements in numerous models. For example, acceptability-rejection models with CNNs values were a percentage of correct classification of 85.30 %, precision of 83.90 %, recall of 86.00 %, and F1 score 84.90 %, as well as AUC-ROC of 0.90. These results assert the its benefits of preprocessing in improving on the future forecast of existing models. Altogether, including new preprocessing schemes together with CASDN opens an effective method for the breast cancer image segmentation. The increases both in segmentation accuracy and in classification performance therefore indicate that this approach could have significant clinical applications. Further developments of the CASDN architecture may be considered in future work, as well as expanding its use in more medical image analysis tasks to help improve diagnosis and treat patients.

## Declaration of competing interest

The authors declare that they have no known competing financial interests or personal relationships that could have appeared to influence the work reported in this paper.

## Data Availability

No data was used for the research described in the article.

## References

[1] Lee H., Park J., Hwang J.Y. (2020). Channel attention module with multiscale grid average pooling for breast cancer segmentation in an ultrasound image. IEEE Trans. Ultrason. Ferroelectr. Freq. Control.

[2] Park J.Y. (2022). Evaluation of breast cancer size measurement by computer-aided diagnosis (CAD) and a radiologist on breast MRI. J. Clin. Med..

[3] Abo-El-Rejal A., Ayman S.E., Aymen F. (2024). Advances in breast cancer segmentation: a comprehensive review. Acadlore Trans. Mach. Learn..

[4] Robertson S., Azizpour H., Smith K., Hartman J. (2018). Digital image analysis in breast pathology—From image processing techniques to artificial intelligence. Transl. Res..

[5] Abhisheka B., Biswas S.K., Purkayastha B. (2023). A comprehensive review on breast cancer detection, classification and segmentation using Deep learning. Arch. Comput. Methods Eng..

[6] Prabaharan G., Dhinakaran D., Raghavan P., Gopalakrishnan S., Elumalai G. (2024). AI-enhanced comprehensive liver tumor prediction using convolutional autoencoder and genomic signatures. Int. J. Adv. Comput. Sci. Appl. (IJACSA).

[7] Vahadane A., Peng T., Sethi A., Albarqouni S., Wang L., Baust M. (2016). Structure-preserving color normalization and sparse stain separation for histological images. IEEE Trans. Med. ImAging.

[8] Jha S., Ahmad S., Arya A., Alouffi B., Alharbi A., Alharbi M., Singh S. (2023). Ensemble learning-based hybrid segmentation of mammographic images for breast cancer risk prediction using fuzzy C-means and CNN model. J. Healthc. Eng..

[9] Wang J., Zheng Y., Ma J., Li X., Wang C., Gee J., Wang H., Huang W. (2023). Information bottleneck-based interpretable multitask network for breast cancer classification and segmentation. Med. Image Anal..

[10] Ramani R., Dhinakaran D., Edwin Raja S., Thiyagarajan M., Selvaraj D. (2024). Integrated normal discriminant analysis in MapReduce for diabetic chronic disease prediction using bivariant deep neural networks. Int. J. Inf. Technol..

[11] Eltalhi S., Kutrani H., Azzuz R., Elbadri F.A.A. (2022). Proceedings of the 2022 IEEE 2nd International Maghreb Meeting of the Conference on Sciences and Techniques of Automatic Control and Computer Engineering (MI-STA).

[12] Fadida-Specktor B. (2018). Preprocessing prediction of advanced algorithms for medical imaging. J. Digit. Imaging.

[13] Rani M., Yadav J., Rathee N., Goyal S. (2022). Proceedings of the 2022 IEEE Delhi Section Conference (DELCON).

[14] De Raad K.B. (2021). Proceedings of the 2021 IEEE 18th International Symposium on Biomedical Imaging (ISBI).

[15] Bozhenko V.V., Tatarnikova T.M. (2023). Proceedings of the 2023 Wave Electronics and its Application in Information and Telecommunication Systems (WECONF).

[16] Almuhaideb S., Menai M.E.B. (2016). Impact of preprocessing on medical data classification. Front. Comput. Sci..

[17] Chalo Alom M.Z., Yakopcic C., Hasan M., Taha T.M., Asari V.K. (2019). Recurrent residual U-net for medical image segmentation. J. Med. ImAging.

[18] Hu H., Qiao S., Hao Y., Bai Y., Cheng R., Zhang W., Zhang G. (2022). Breast cancer histopathological images recognition based on two-stage nuclei segmentation strategy. PLoS One.

[19] Han Z., Wei B., Zheng Y., Yin Y., Li K., Li S. (2017). Breast cancer multi-classification from histopathological images with structured deep learning model. Sci. Rep..

[20] Krishnakumar B., Kousalya K. (2023). Optimal trained deep learning model for breast cancer segmentation and classification. Inf. Technol. Control.

[21] Lee H., Park J., Hwang J.Y. (2020). Channel attention module with multiscale grid average pooling for breast cancer segmentation in an ultrasound image. IEEE Trans. Ultrason. Ferroelectr. Freq. Control.

[22] Mustafa M., Rashid N.A.O., Samad R. (2014). Proceedings of the 2014 IEEE Conference on Biomedical Engineering and Sciences (IECBES).

[23] Qin C., Wu Y., Zeng J. (2022). Joint transformer and multi-scale CNN for DCE-MRI breast cancer segmentation. Soft Comput..

[24] Zarei M., Rezai A., Falahieh Hamidpour S.S. (2021). Breast cancer segmentation based on modified Gaussian mean shift algorithm for infrared thermal images. Comput. Methods Biomech. Biomed. Eng. Imaging Vis..

[25] Samudrala S., Mohan C.K. (2024). Semantic segmentation of breast cancer images using DenseNet with proposed PSPNet. Multimed. Tools Appl..

[26] Wu Y., Zhou Y., Saveriades G., Agaian S., Noonan J.P., Natarajan P. (2013). Local Shannon entropy measure with statistical tests for image randomness. Inf. Sci..

[27] Dong N., Kampffmeyer M., Liang X., Wang Z., Dai W., Xing E. (2018).

[28] Qin C., Wu Y., Zeng J. (2022). Joint transformer and multi-scale CNN for DCE-MRI breast cancer segmentation. Soft Comput..

[29] Hu H., Qiao S., Hao Y., Bai Y., Cheng R., Zhang W., Zhang G. (2022). Breast cancer histopathological images recognition based on two-stage nuclei segmentation strategy. PLoS One.

[30] Wang X., Tang L., Mou H., Peng S., Zhu X., Lai X. (2023). Proceedings of the 2023 7th International Conference on Big Data and Internet of Things (BDIOT '23).

[31] Zeebaree D.Q., Haron H., Abdulazeez A.M., Zebari D.A. (2019). Proceedings of the 2019 International Conference on Advanced Science and Engineering (ICOASE).

[32] Hu Y.H., Lin W.C., Tsai C.F., Ke S.W., Chen C.W. (2015). An efficient data preprocessing approach for large-scale medical data mining. Technol. Health Care.

